# Three-dimensional Structure of Victorivirus HvV190S Suggests Coat Proteins in Most Totiviruses Share a Conserved Core

**DOI:** 10.1371/journal.ppat.1003225

**Published:** 2013-03-14

**Authors:** Sarah E. Dunn, Hua Li, Giovanni Cardone, Max L. Nibert, Said A. Ghabrial, Timothy S. Baker

**Affiliations:** 1 Department of Chemistry and Biochemistry, University of California San Diego, La Jolla, California, United States of America; 2 Department of Plant Pathology, University of Kentucky, Lexington, Kentucky, United States of America; 3 Department of Microbiology and Immunobiology, Harvard Medical School, Boston, Massachusetts, United States of America; 4 Division of Biological Sciences, University of California San Diego, La Jolla, California, United States of America; The Scripps Research Institute, United States of America

## Abstract

Double-stranded (ds)RNA fungal viruses are currently assigned to six different families. Those from the family *Totiviridae* are characterized by nonsegmented genomes and single-layer capsids, 300–450 Å in diameter. Helminthosporium victoriae virus 190S (HvV190S), prototype of recently recognized genus *Victorivirus*, infects the filamentous fungus *Helminthosporium victoriae* (telomorph: *Cochliobolus victoriae*), which is the causal agent of Victoria blight of oats. The HvV190S genome is 5179 bp long and encompasses two large, slightly overlapping open reading frames that encode the coat protein (CP, 772 aa) and the RNA-dependent RNA polymerase (RdRp, 835 aa). To our present knowledge, victoriviruses uniquely express their RdRps via a coupled termination–reinitiation mechanism that differs from the well-characterized Saccharomyces cerevisiae virus L-A (ScV-L-A, prototype of genus *Totivirus*), in which the RdRp is expressed as a CP/RdRp fusion protein due to ribosomal frameshifting. Here, we used transmission electron cryomicroscopy and three-dimensional image reconstruction to determine the structures of HvV190S virions and two types of virus-like particles (capsids lacking dsRNA and capsids lacking both dsRNA and RdRp) at estimated resolutions of 7.1, 7.5, and 7.6 Å, respectively. The HvV190S capsid is thin and smooth, and contains 120 copies of CP arranged in a “T = 2” icosahedral lattice characteristic of ScV-L-A and other dsRNA viruses. For aid in our interpretations, we developed and used an iterative segmentation procedure to define the boundaries of the two, chemically identical CP subunits in each asymmetric unit. Both subunits have a similar fold, but one that differs from ScV-L-A in many details except for a core α-helical region that is further predicted to be conserved among many other totiviruses. In particular, we predict the structures of other victoriviruses to be highly similar to HvV190S and the structures of most if not all totiviruses including, Leishmania RNA virus 1, to be similar as well.

## Author Summary

Of the known dsRNA fungal viruses, the best characterized is Saccharomyces cerevisiae virus L-A (ScV-L-A), prototype of the genus *Totivirus*, family *Totiviridae*. Until the current study, there have been no subnanometer structures of dsRNA fungal viruses from the genus *Victorivirus*, which is the largest in family *Totiviridae*. The 3D cryo-reconstruction presented here of prototype victorivirus Helminthosporium victoriae virus 190S (HvV190S) approaches 7-Å resolution and shows the asymmetric unit of the capsid is a dimer comprising two, chemically identical coat-protein subunits organized in a so called “T = 2” lattice. These HvV190S subunits have a similar fold, but one that differs from ScV-L-A in many details except for a core α-helical region that is further predicted to be conserved among many other totiviruses. In particular, we predict the structures of other victoriviruses to be highly similar to HvV190S and the structures of most if not all totiviruses, including Leishmania RNA virus 1, to be similar as well.

## Introduction

The encapsidated, double-stranded (ds)RNA viruses infect a wide range of hosts including bacteria, plants, fungi, insects, and humans and other vertebrates [1], [2]. Viruses that infect and replicate in fungi (mycoviruses) have no known natural vectors and are spread vertically or horizontally by intracellular means [3], [4]. Despite lacking an extracellular phase [4], mycoviruses are successfully disseminated through all major groups of fungi [5], [6]. As such, fungal viruses have been touted as potentially beneficial, biological control agents of pathogenic fungi that infect economically important agricultural crops. This has added significance because fungicides in current use pose health hazards and environmental risks [4].

Encapsidated dsRNA mycoviruses are currently classified in six families. Each member of family *Totiviridae* has a nonsegmented genome, but members of families *Partitiviridae*, *Megabirnaviridae*, *Chrysoviridae*, *Quadriviridae*, and *Reoviridae* have genomes comprising 2, 2, 4, 4, and 11–12 segments, respectively [4], [7]–[9]. The capsids of all these viruses have an overall, spherical morphology and are constructed from an icosahedrally symmetric arrangement of one or more capsid proteins. Most of these capsids are single-shelled and range in diameter from ∼300 to ∼450 Å, with the exception of the larger, double-shelled reoviruses (600–850 Å) [4]. All the single-shelled capsids (except those of chrysoviruses), as well as the inner capsids of reoviruses, consist of 120, chemically identical subunits arranged in a so-called “T = 2”, icosahedral lattice [10]. In addition, all dsRNA viruses, including those that infect fungi, must package one or more virally encoded RNA-dependent RNA polymerase (RdRp) molecules that replicate and transcribe the viral genome, since dsRNA cannot function as mRNA [1].

Totiviruses are the simplest encapsidated dsRNA mycoviruses in containing a single genome segment that encodes only two proteins: coat protein (CP) and either RdRp or a CP/RdRp fusion [9]. Members of family *Totiviridae* therefore provide a simple model system for studying mycoviruses, as well as dsRNA viruses more generally. Family *Totiviridae* comprises five current genera: *Totivirus, Victorivirus, Giardiavirus, Leishmaniavirus, and Trichomonasvirus* [9], [11]. Members of the first two infect fungi, whereas the others infect protozoa [9]. Infectious myonecrosis virus (IMNV), a tentative totivirus that infects shrimp [12] is currently unclassified, as are more recently discovered, tentative totiviruses that infect insects and fish [13]–[16].

Of the five genera in family *Totiviridae*, genus *Victorivirus* is currently largest, with 14 members and probable members [17], [18]. Victoriviruses differ from the four other genera in their manner of RdRp translation (see below) and in the C-terminal sequences of their CPs. Victorivirus CPs range in mass from 77 to 83 kDa (746–780 aa) and, to our current knowledge, share the unique feature of having a C-terminal region enriched in Ala, Gly, and Pro residues. The combined percentage of these residues in the C-terminal region of HvV190S is 52%, which differs considerably compared to the 24%, 22%, 18% and 17% present in similarly-sized C-terminal regions (130-aa stretches) of representatives from the *Totiviridae* genera *Leishmaniavirus* (LRV1-1), *Totivirus* (ScV-L-A), *Trichomonasvirus* (TVV1), and *Giardiavirus* (GLV), respectively. Unlike some viruses in the other genera, no confirmed satellite dsRNAs, and consequently no satellite-encoded killer toxins, are known to be associated with victoriviruses. Host genes, however, are known to be upregulated during infections by one victorivirus, Helminthosporium victoriae virus 190S (HvV190S) [18], [19]. Those genes include the *victoriocin* or *vin* gene that encodes a broad-spectrum, antifungal protein [20] and a gene that encodes the multifunctional Hv-p68 protein, which exhibits protein kinase, alcohol oxidase, and RNA-binding activities [21], [22].

HvV190S was first discovered in 1978 in the filamentous fungus *Helminthosporium victoriae* (*H. victoriae*, telomorph: *Cochliobolus victoriae*), the causal agent of Victoria blight of oats [23]. The 5179-bp HvV190S genome includes two large, slightly overlapping, open reading frames (ORFs) that encode the CP (772 aa; calculated mass, 81 kDa) and the RdRp (835 aa; calculated mass, 92 kDa) [17], [24]. Notably, virus-infected *H. victoriae* exhibits symptoms typical of a disease phenotype [4], which is unusual for dsRNA mycoviruses, in that most of them do not cause symptoms in their respective hosts. HvV190S may therefore provide an additional, useful model system for studying mycoviruses that have debilitating effects on their hosts [4].

HvV190S and likely other members of the genus *Victorivirus* [9] are unlike most other members of family *Totiviridae* in the mechanism by which their RdRps are translated. Members of most genera express their RdRps as a fusion protein with CP consequent to ribosomal frameshifting [25]. HvV190S, in contrast, expresses its RdRp as a separate, nonfused protein, using a coupled termination/reinitiation (stop/restart) strategy that involves an AUGA motif in which CP terminates at UGA and translation restarts at AUG to make RdRp. [26], [27]. The RNA sequence requirements for the stop/restart mechanism include a 32-nt region that contains a predicted pseudoknot and lies close to the downstream AUGA motif [27].

Though the HvV190S genome contains just two ORFs, SDS-PAGE of purified HvV190S virions reveals the presence of three forms of CP, which have been shown by peptide analysis to be closely related and named p88, p83, and p78 to reflect their respective M_r_ values [9]. Expression in both bacterial and eukaryotic systems has shown p88 is the primary translation product, and p83 and p78 are derived from p88 via proteolytic processing at the C-terminus. Also, p88 and p83 are phosphorylated, but p78 is not [28], [29]. Purified HvV190S virions include 190S-1 and 190S-2 forms, which differ slightly in sedimentation rate and capsid composition [9], [28]. When separated by two cycles of sucrose density gradient centrifugation, samples of 190S-1 contain similar amounts of p88 and p83 whereas samples of 190S-2 contain similar amounts of p88 and p78 (Figure S1A). The ratio of 190S-1 to 190S-2 particles varies in fungal cultures according to age, with 190S-2 predominating in virion preparations from 14-day or older cultures [29]. However, regardless of culture age, the yield of the separated 190S-1 and 190S-2 is always very low owing to significant losses during the separation protocol [29] and thus is not suitable for structure studies.

HvV190S, like Saccharomyces cerevisiae virus L-A (ScV-L-A, prototype of genus *Totivirus*) and presumably all other members of family *Totiviridae*, has a “T = 2” arrangement of CP subunits [17], [25]. Currently, the 3D structures of only a few dsRNA mycoviruses have been determined, and the only high-resolution crystal structure is that of ScV-L-A [30]. All other mycovirus structures have been examined by means of transmission electron cryomicroscopy (cryoEM) and 3D image reconstruction methods. These include structures of Ustilago maydis virus H1, another current member of genus *Totivirus* [31]; three partitiviruses, Penicillium stoloniferum virus F [32], Penicillium stoloniferum virus S [10], and Fusarium poae virus 1 [33]; and two chrysoviruses, Penicillium chrysogenum virus [34], [35] and Cryphonectria nitschkei chrysovirus 1 [36].

Here we used cryoEM and 3D image reconstruction to examine the structures of HvV190S virions and two types of HvV190S virus-like particles (VLPs), called VLP_C+_ and VLP_C_. VLP_C+_ lack the dsRNA genome, whereas VLP_C_ lack both the genome and the RdRp. The 3D structure of HvV190S VLP_C_ was determined by cryoEM in an earlier study at ∼14-Å resolution [37]. We now report the 3D structures of HvV190S virions and both types of VLPs, all at subnanometer resolution (∼7–8 Å). From these data, we have been able to define the molecular envelopes of the two CP monomers, “A” and “B”, in each asymmetric unit of the “T = 2” capsid. These monomers are morphologically similar to each other as well as to the corresponding monomers of ScV-L-A. However, except for two α-helices in the subunit core and a large β-sheet on one side of the subunit, the folds of HvV190S and ScV-L-A CP are quite different. Comparisons of the predicted secondary structures of several different totiviruses indicate that this helix-rich core may be a highly conserved feature among all totiviruses and perhaps other dsRNA mycoviruses as well.

## Results

### Preparation of HvV190S samples

**Figure 1:**
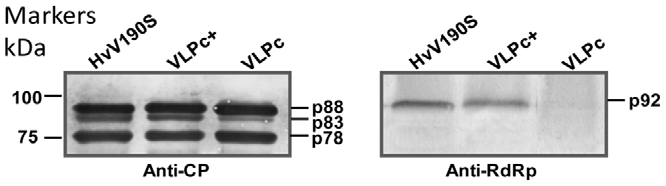
Western Blot analysis of the capsid proteins and RdRp of the HvV190S virion and VLPs. Purified HvV190S virions and VLP_c+_ and VLP_c_ were subjected to immunoblot analysis using a CP-specific antiserum (left panel) or an RdRp-specific antiserum (right panel).

HvV190S particles were purified from a naturally infected culture of *H. victoriae* strain A-9. VLP_C+_ and VLP_C_ were purified from virus-free strain B-2ss, which was transformed with p190S [27] containing a full-length cDNA of HvV190S dsRNA (for VLP_C+_) or with p190S containing only the CP ORF (for VLP_C_). All *H. victoriae* cultures were grown for 14 days prior to harvest and purification. Western blot analysis, using a CP-specific antiserum, confirmed that all preparations of the three particle types (virion, VLPc_+_ and VLPc) contained the three related capsid proteins p88, p83, and p78 (Figure 1, left panel). The higher relative proportion of p78 compared to p83 reflects the fact that the 190S-2 form (Figure S1A) predominates in 14-day cultures ([29], also see Introduction). A second Western blot, using an RdRp-specific antiserum, confirmed that only virions and VLP_C+_ contained RdRp (Figure 1, right panel).

### CryoEM of HvV190S virions and VLPs

**Figure 2:**
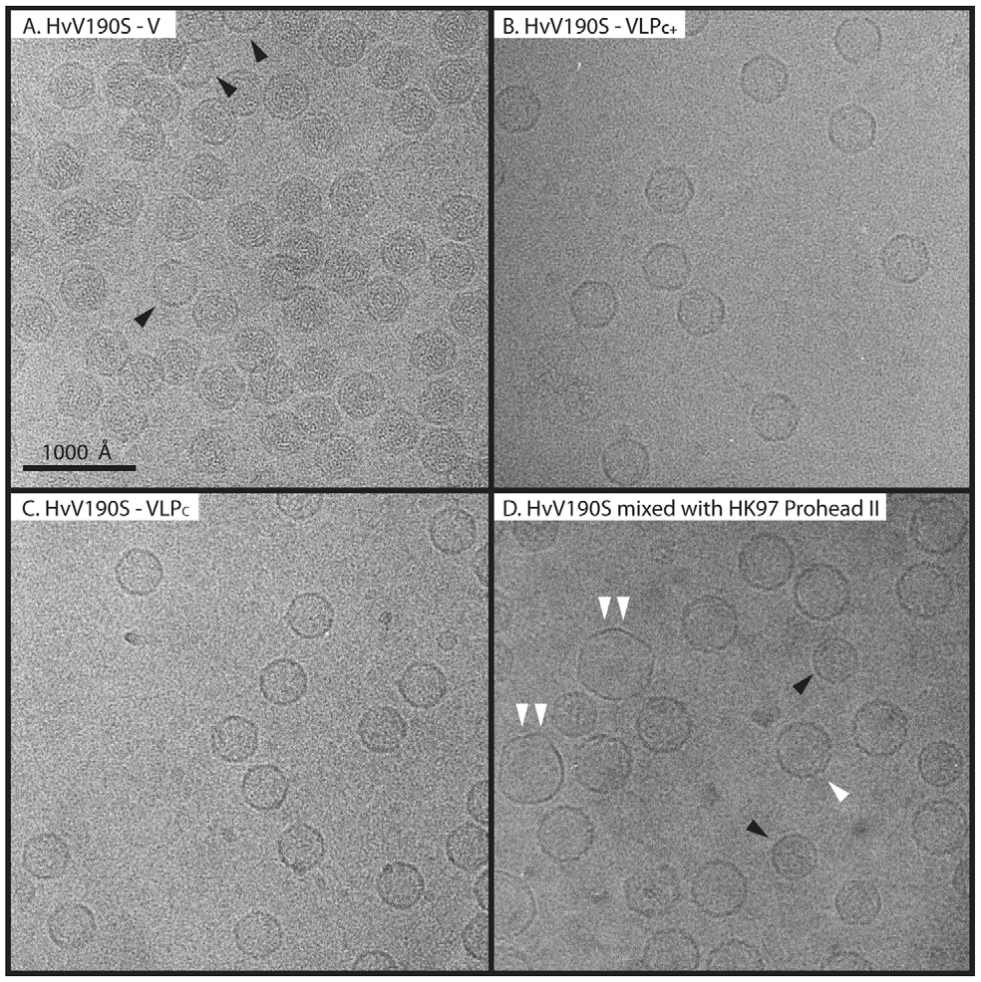
Electron micrographs of unstained, vitrified samples of HvV190S virions and VLPs. Representative cryo-micrographs of four separate samples are shown: (**A**) HvV190S virions; (**B**) VLP_C+_; (**C**) VLP_C_; and (**D**) HvV190S virions mixed with HK97 prohead II. Three particles in the virion sample (A, black arrowheads) appear empty and presumably have lost their genomes. In (D), the HK97 prohead II (white arrowhead) is clearly distinct from the smaller HvV190S virions (black arrowheads). The two largest particles in the field of view are HK97 particles that have spontaneously expanded (double arrowhead). The contrast in all panels has been adjusted to improve visibility of each sample.

Cryo-micrographs of the three particle types show that most of the HvV190S virions and VLPs are roughly the same size and shape, with relatively smooth, featureless profiles and no obvious protrusions (Figure 2). Most of the virions exhibit approximately uniform density throughout, as expected for particles that contain genome. A few particles in this sample appear to be empty or partially empty (labeled in Figure 2A), and those likely are virions that have lost genome. The fraction of such particles in virion preparations increases with time, as does the fraction of aggregated particles (not shown), and indicates that HvV190S virions are relatively unstable. All VLP samples contained particles with little density inside the confines of a thin capsid (Figure 2 B, C). The absolute size of the HvV190S virions was determined by recording their cryoEM images in samples mixed with HK97 prohead II particles (Figure 2D). The crystal structure of the latter is known [38], and hence these make an excellent, internal standard for calibrating microscope magnification (see Materials and Methods).

### 3D Reconstructions of HvV190S virion and VLPs

**Figure 3:**
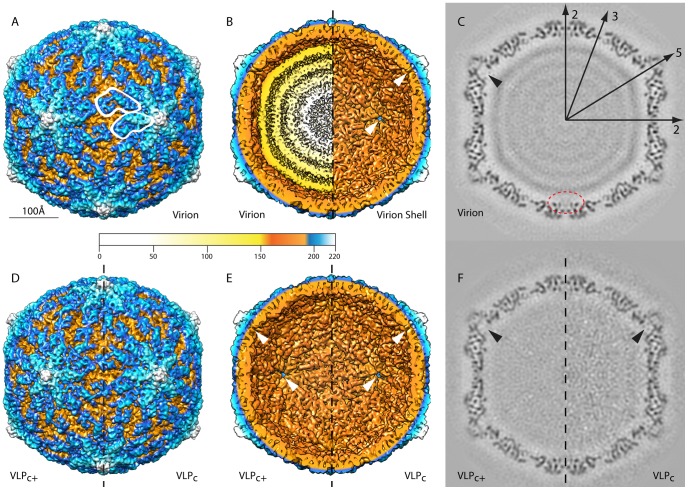
Cryo-reconstructions of HvV190S particles. (**A**) Radial, color-coded, surface view along a twofold axis of the HvV190S virion reconstruction. A pair of similar features (outlined in white) correspond to raised portions of the two capsid subunits in one asymmetric unit of the “T = 2” capsid. (**B**) Same as (A), with the front half of the density map eliminated to show the particle interior (left) and with the genome density computationally removed to show the inner surface of the capsid (right). An innermost, fifth shell of RNA on the left side of the panel is not visible because its intensity level is close to that of noise in the density map, and the threshold used to render the map was set to a value slightly higher than this; however, a radial density plot of the virion density map (Figure S3) suggests the presence of this fifth shell of RNA density. White arrowheads point to two different views of the channel located at each five-fold axis. (**C**) Planar, equatorial density projection (one pixel or ∼1.07 Å thick) of the HvV190S virion cryo-reconstruction, with features of highest and lowest density depicted in black and white, respectively. Representative two-, three-, and fivefold axes of symmetry that lie in the equatorial plane are indicated. The black arrowhead points to the channel at one fivefold axis and the dashed red oval encircles the density features that span the space near the I2 axis between the capsid and genome. (**D**) Same as (A), for the VLP_C+_ (left half) and VLP_C_ (right half). (**E**) Inner surfaces, as in (B), for the VLP_C+_ on the left and the VLP_C_ on the right. (**F**) Same as (C), for the VLP_C+_ (left) and VLP_C_ (right). The scale bar in (A) is the same for all panels and the color bar specifies radii in Å units.

Cryo-reconstructions of HvV190S virions, VLP_c+_, and VLP_C_, at estimated resolutions of 7.1, 7.5, and 7.6 Å, were computed from 20,904, 16,046, and 6,294 particle images, respectively (Table 1; Figures 3 and S2). When the density maps are rendered at a threshold level set according to the expected mass of the capsid and are color coded to accentuate small radial differences, the outer surfaces of all three capsids appear essentially identical, with a complex distribution of features (Figure 3 A, D). The inner surfaces are also virtually identical and complex (Figure 3 B, E). Each capsid has a relatively smooth, spherical outer profile that is interrupted only by small protrusions at the twelve icosahedral fivefold (I5) vertices where the maximum diameter reaches 462 Å. The outer surface of the capsid drops to a minimum diameter of 356 Å at the icosahedral threefold (I3) and ∼368 Å at the icosahedral twofold (I2) axes. The inside surface of the capsid approximates a sphere of ∼327 Å minimum diameter except for small, mushroom-shaped cavities that extend radially outward along the I5 axes (Figure 3 B, E, white arrowheads; Figure 3 C, F, black arrowheads). The average thickness of the capsid is ∼35 Å but drops to a minimum of just 6 Å at the I3 axes (Figure 3 C, F). Radial density plots of the cryo-reconstructions (Figure S3) show that the capsid region is centered near radius 183 Å in each. The internal features of all three capsids also appear virtually identical, as illustrated in central, equatorial sections of the density maps (Figure 3 C, F). However, close comparisons of these maps reveal the presence of subtle changes that are not easily detected by eye (see Discussion and Movie S1).

**Table 1:** 3D image reconstruction statistics for HvV190S.

Specimen	Micrographs^1^	Particles^2^	Defocus^3^	Resolution^4^
**Virion**	176	20,904	0.72–3.80 µm	7.1 (6.8) Å
**VLP_C+_**	289	16,046	0.69–3.89 µm	7.5 (7.1) Å
**VLP_C_**	100	6,294	0.43–3.88 µm	7.6 (7.2) Å

1 Number of micrographs selected for image processing.

2 Number of particle images included in each 3D reconstruction.

3 Range of objective lens underfocus settings.

4 Estimate of resolution achieved in 3D reconstruction based on FSC_0.5_ and FSC_0.143_ criteria [56]. See also Figure S2.

The HvV190S virion reconstruction shows the genome packed in four or five concentric, roughly spherical shells (Figure 3 B, C; Figure S3), each with a maximum average density weaker than the capsid. Also, the level of organization within each shell diminishes with decreasing radius as indicated by progressively lower average density. The average spacing of these shells is ∼30 Å, which compares favorably with genome spacings observed in ScV-L-A and other dsRNA mycoviruses [10], [12], [30], [32]. A gap of ∼17 Å separates most of the inner wall of the capsid from the outermost shell of dsRNA. This includes the regions near the I5 axes where the outermost genome shell is thickest and bulges outwards at the base of each cavity (Figure 3 B, C). The inner surface of the capsid extends to its lowest radius (156 Å) near the I2 axes, and densities spanning the sub-capsid space at those sites suggest contacts with genome (Figure 3C, dashed red oval).

Individual subunits in the HvV190S capsid are hard to discern by direct inspection of the density maps (Figure 3 A, D). However, morphological features repeated 120 times on the capsid surface, consistent with a “T = 2” icosahedral arrangement as found in other dsRNA viruses [10], [32], [33], [39], can be identified upon careful examination. These “T = 2” structures consist of 60 asymmetric dimers, each containing two chemically identical monomers that occupy nonidentical environments, “A” and “B”, in the icosahedron. A-subunits cluster around the I5 axes, and B-subunits around the I3 axes. Close analysis of all three HvV190S cryo-reconstructions showed that at higher radii the capsid surface exhibits a staggered arrangement of two, similarly shaped features in nonidentical environments (Figure 3 A, D). This exposed portion of each subunit contains a narrow “tip” on the proximal side (near the I5 axis) and a hook-like “anchor” on the distal side (near the I2 axis). The tips of the A- and B-subunits lie respectively ∼9 and ∼47 Å away from the I5 axis, and the anchor of each A-subunit faces the anchor of a B-subunit from an adjacent capsid vertex region in a quasi-twofold arrangement.

**Figure 4:**
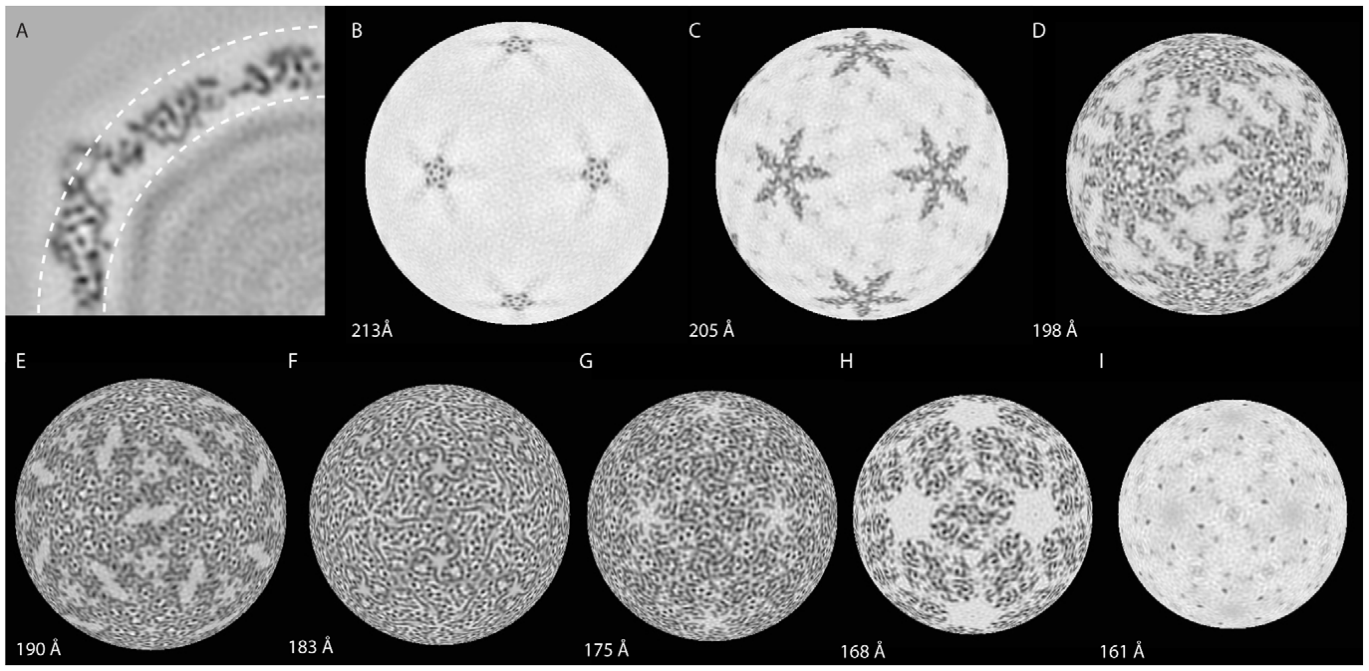
Radial density projections of the HvV190S virion. (**A**) Enlarged view of upper left quadrant shown in Figure 3C with arcs drawn at radii corresponding to the density projections shown in panels B (high radius) and I (low radius). (**B–I**) Spherical, one-pixel (∼1.07 Å) thick sections from the HvV190S virion cryo-reconstruction are shown at progressively lower radii as indicated in each panel, with highest and lowest density features depicted in black and white, respectively. Panel F corresponds to the radius (r = 183 Å) where the inter-subunit interactions are most prevalent and where the capsid-region densities are centered (see Figure S3).

The HvV190S capsid, viewed in equatorial sections, is composed of numerous punctate and linear density features (Figure 3 C, F), consistent with the presence of α-helices that lie perpendicular to or in the plane of the sections, respectively. Different, yet complementary views of the HvV190S capsid seen in spherical density projections (Figure 4) show that these same types of features occur throughout the capsid at all radii. They also illustrate that a large portion of the A-subunit outer surface lies at higher radius than any portion of the B-subunit (Figure 4C). The anchors of the A- and B-subunits lie at about the same radius (Figure 4D), which indicates that the A-subunit is tilted relative to B. At lower radii, density features form a tightly interwoven, complex meshwork (Figure 4 E–H), making it difficult to assign specific densities unambiguously to A or B. The highest packing density in the HvV190S capsid appears at radius 183 Å (Figure 4F), consistent with the radial density plot mentioned above.

To define more precisely how the A- and B-subunits are oriented relative to each other, we measured the radial positions of three specific structural features in the HvV190S virion reconstruction. First, the proximal tip of B sits ∼18 Å lower than that of A. Second, the distal tip of the B anchor, which lies close to the I3 axis, is ∼7 Å higher than that of the A anchor. These different radial heights of the proximal and distal tips of A and B demonstrate that the two subunits are tilted relative to one another, approximately along a line between neighboring I5 and I3 axes. The two subunits are also tilted relative to one another in the perpendicular direction (i.e., along a line between neighboring I3 and I2 axes), which positions the portion of the B anchor that lies close to the I2 axis at about the same radius as that of A. This relative arrangement of A- and B-subunits in virions is slightly altered in the VLPs. In both VLPs, both A and B are less tilted than their counterparts in virions, by 1° and 0.5°, respectively. Nevertheless, their proximal tips maintain a radial separation of ∼18 Å, and it follows that the A- and B-subunits remain tilted relative to one another in the VLPs. Hence, in all three HvV190S capsids, the A- and B-subunits adopt a staggered arrangement both in radius and in proximity to the I5 axis, though they do exhibit minor changes in their relative alignments.

### Segmentation of the HvV190S capsid

Given the difficulties inherent in assigning densities directly to the A- and B-subunits of the HvV190S capsid, we next tried to use the atomic structure of the A–B dimer of ScV-L-A [30] as a rigid body to model the HvV190S structure and delineate subunit boundaries. However, this process failed, primarily because the CPs of these two totiviruses differ markedly in size (680 vs. 772 aa for ScV-L-A and HvV190S, respectively) and sequence (see below). An initial assessment of the fit of the ScV-L-A dimer into the HvV190S virion density map gave no clear-cut indication of the monomer boundaries and also, as suggested previously [37], showed no obvious evidence of similar folds of the HvV190S and ScV-L-A CPs.

**Figure 5:**
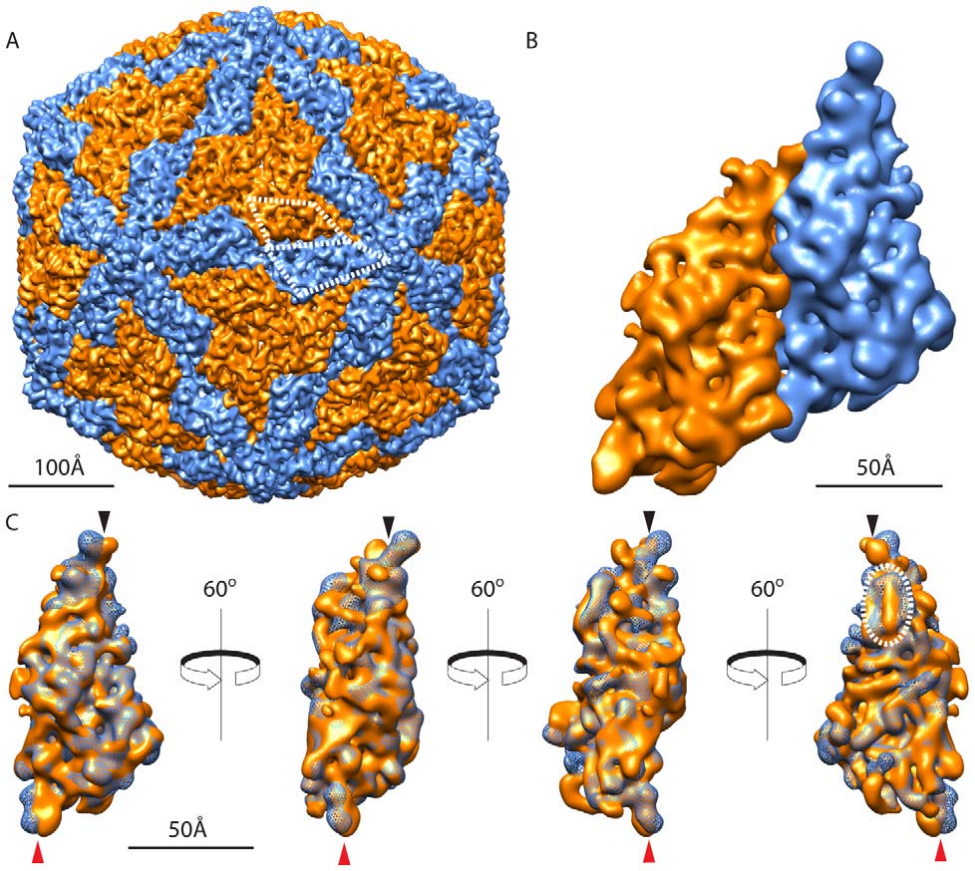
Segmentation of the HvV190S virion capsid. (**A**) View along a twofold axis of the segmented capsid with the A- and B-subunits colored in blue and orange, respectively. Each of the subunits in one asymmetric dimer is outlined with a quadrilateral polygon. (**B**) Enlarged view of one asymmetric dimer. (**C**) Superposition of segmented A- and B-subunits shown in four different orientations, beginning with a view as seen from outside the particle (identical to the A subunit shown in panel B) and ending with a view from inside the capsid (far right). Predominant differences occur at the proximal (black arrowheads) and distal (red arrowheads) tips of the two subunits and in a pair of parallel helices (dashed white oval) that follow a curved trajectory in the A-subunit but a straighter, laterally displaced trajectory in the B-subunit.

Without ScV-L-A as a guide, we turned to using an *ab initio* approach to segment out each subunit in the HvV190S virion reconstruction (see Materials and Methods). This method relies on two principal assumptions: that the A- and B-subunits have compact, similarly folded structures and that a full, 120-subunit capsid model generated from the segmented A–B dimer would uniquely account for all non-genome density in the cryo-reconstruction. Results of the segmentation procedure showed that both subunits, as viewed from outside the capsid, have similar, asymmetric profiles that resemble a convex quadrilateral (Figure 5 A, B). These quadrilaterals have dimensions of ∼74–97 Å on opposing long sides and ∼56–67 Å on opposing short sides. The longest dimensions of the A- and B-subunits are about 125 and 124 Å, respectively. Side views (Figure 5C) show them to vary in thickness from about 34 to 44 Å, which is consistent with the equatorial section views (Figure 3 C, F) and the fact that the long axes of the subunits adopt near-tangential orientations in the capsid. Overall, the 3D shapes of the A and B subunits are quite similar and superimpose well (Figure 5C), indicating that each monomer has about the same tertiary structure. Two of the largest and most obvious deviations occur at the proximal (Figure 5C, black arrowheads) and distal (Figure 5C, red arrowheads) tips of the two subunits. Additional large differences are seen in corresponding regions on the interior side of the subunits (encircled in the rightmost view in Figure 5C), where a pair of parallel features ascribed to a set of α-helices follows either a curved (A-subunit) or straighter (B-subunit) path.

As seen in a surface view (Figure 5A), extensive intersubunit contacts produce a nearly impenetrable shell. However, close inspection of all three HvV190S cryo-reconstructions reveals two small, solvent-accessible pores at each I2 axis and three, even smaller pores close to each I3 axis. The presence of these pores is confirmed by visual inspection of the grayscale density map, though their sizes and shapes cannot be determined precisely because they are influenced by the final resolution of the map (the actual size of a pore or channel can only be measured accurately and reliably in maps at atomic or near-atomic resolution). Of note, the pores in both VLPs appear smaller than their counterparts in virions.

At highest radii in the capsid, the proximal tips of five adjacent A-subunits form a unique set of interactions at the vertices (I5 axes) of the icosahedron. The only other A–A contact on the capsid surface occurs via the distal tips of two A subunits across each I2 axis, but this contact is much less extensive than those around each I5 axis. B–B contacts at the capsid surface are extensive but occur only among the B subunits that surround each I3 axis. The most extensive of all contacts at the capsid surface, however, occur between A- and B-subunits, such that each A or B is mostly surrounded by three distinct B- or A-subunits, respectively.

The nonidentical environments that the A- and B-subunits occupy in the capsid are quite striking, as illustrated by two examples. First, the proximal tip of the A-subunit interacts with two, symmetry-related tips of neighboring A-subunits around the capsid vertex, whereas the proximal tip of the B-subunit interacts with distinct surfaces of two adjacent A-subunits. Second, the distal tip of each B-subunit interacts with distinct surfaces of two adjacent B-subunits around each I3 axis, whereas the distal tip of each A-subunit interacts with the distal tip of another A-subunit across each I2 axis and also with a distinct portion of an adjacent B-subunit.

### The CPs of HvV190S and ScV-L-A have similar shapes but different secondary and tertiary structures

**Figure 6:**
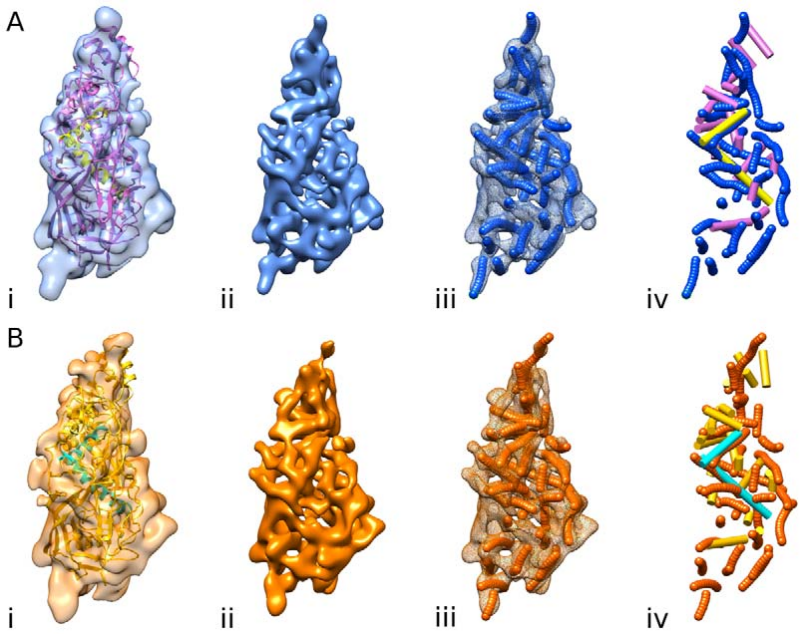
Fit of ScV-L-A CP subunit crystal structures into HvV190S reconstruction and prediction of α-helices in HvV190S. (**A**) Segmented density map of the HvV190S virion A-subunit (blue), oriented as in Figure 5B. (**B**) Segmented density map of the HvV190S virion B-subunit (orange), oriented as in the leftmost sub-panel in Figure 5C. (**i**) ScV-L-A A-subunit and B-subunit ribbon models in purple and gold, respectively, fit as rigid bodies into the segmented density maps of the HvV190S A- and B-subunits. A pair of helices, highlighted in yellow (panel A) and green (panel B) are the only helices in ScV-L-A (aa 123–140 for the helix near the top and aa 358–383 for the longer helix) that superimpose with corresponding tubular density features in each HvV190S subunit map (see text). (**ii**) Surface rendering of the HvV190S subunits represented at higher threshold (8σ vs. 2σ). (**iii**) Mesh rendering of the HvV190S subunits along with α-helices (strings of small spheres) predicted by the VolTrac algorithm [66]. (**iv**) Same as (iii) but with the mesh density removed and the helices of ScV-L-A added for comparison and shown as cylinders using the same color scheme as in (i). Also see Movie S2, which gives an animated view of panel A.

Having reliable estimates of the segmented volumes of the HvV190S A- and B-subunits, but insufficient resolution in any of the cryo-reconstructions to trace the peptide backbone of either CP, we revisited whether the ScV-L-A crystal structure could guide our analysis and interpretation of the HvV190S data. The CPs of both viruses do in fact have similar, quadrilateral-shaped morphologies, and rigid-body, quantitative fitting of the individual ScV-L-A A- and B-subunit structures into the corresponding, segmented density volumes of the HvV190S virion reconstruction revealed several regions of unassigned densities in HvV190S (Figure 6 Ai, Bi). These regions include the proximal tip of the A-subunit and a portion of the proximal tip of B, the entire distal tips of both subunits, and a large portion of the long edge of both subunits (right half of each subunit volume seen in Figure 6). In addition, there is unassigned density on the shorter side of the B-subunit (left half in Figure 6B). The presence of such unassigned densities is consistent with the HvV190S CP being 92 aa longer than ScV-L-A CP and also arises because the ScV-L-A models do not include the C-terminal 29 aa in each subunit since they were disordered in the crystal structure [30]. Close inspection of the data showed that most secondary-structure elements in the ScV-L-A subunit models failed to match features in the map for either HvV190S subunit. Nonetheless, three isolated segments in each fitted ScV-L-A model did correlate well with density features in the HvV190S maps. These included two α-helices (helix 5, aa 120–139, and helix 13, aa 358–383) and an antiparallel β-sheet comprising three main strands (aa 26–38, 41–54, and 587–600) and three small strands (aa 302–304, 487–491, and 604–606). The α-helices (colored yellow in subunit A and green in B; Figure 6 Ai, Bi, respectively) constitute a portion of the central core of each ScV-L-A subunit and correspond closely in size and location to tubular density features in each HvV190S subunit (more apparent in maps contoured at higher density thresholds; Movie S2). For comparison, we annotated via an automatic procedure (see Materials and Methods) the location and length of possible α-helices in the segmented density (Figure 6 Aiii, Biii), and found agreement only with the above two mentioned helices of ScV-L-A (Figure 6 Aiv, Biv). An additional correlation was observed between the β-sheet in each ScV-L-A subunit and a large, plate-like density feature located near the distal end and shorter side of each HvV190S subunit (Figure 6 Ai, Bi).

**Figure 7:**
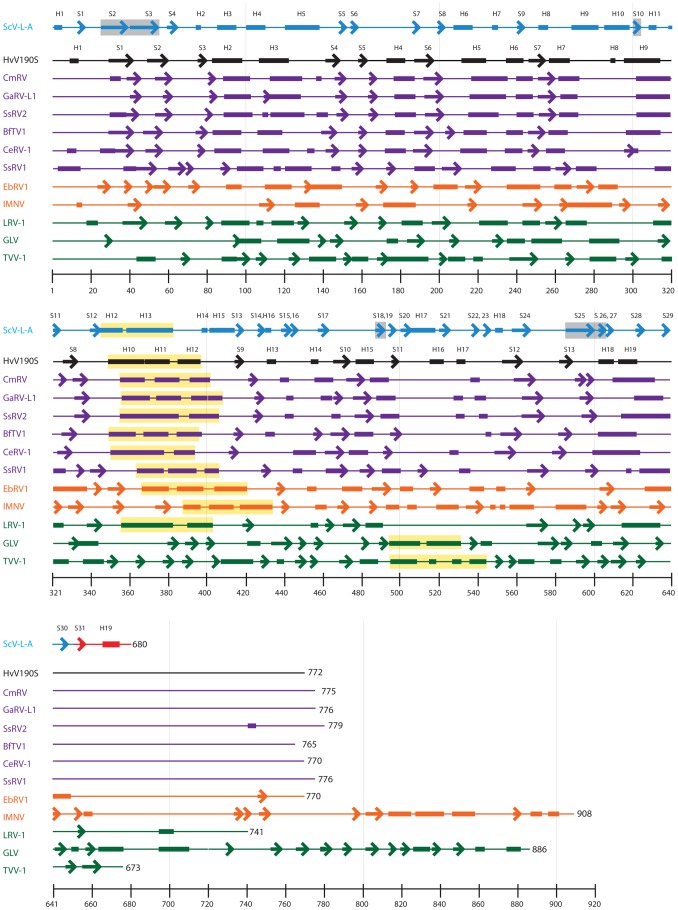
Secondary structure of the ScV-L-A capsid protein and predicted structures of the CPs for HvV190S and eleven other viruses. For ScV-L-A, the secondary structure for the N-terminal 651 aa of the CP (cyan) is derived from the crystal structure (PDB ID 1M1C, [30]) and is predicted for the C-terminal 29 aa (red). The α-helix, β-strand, and random coil segments are represented schematically as rectangles, arrows, and lines, respectively, for ScV-L-A and all predicted structures. The six strands that comprise the prominent β-sheet in ScV-L-A (see text) are highlighted in grey. All secondary-structure predictions were made with the PSIPRED server (see Materials and Methods). The predicted HvV190S secondary structure is rendered in black and six other, representative members of the *Victorivirus* genus are colored purple. Representative members of the *Giardiavirus*, (GLV), *Leishmaniavirus* (LRV-1), and *Trichomonasvirus* (TvV-1) genera are colored green and two unclassified viruses (EbRV1 and IMNV) are colored orange. The stretch of generally two or three adjacent helices predicted in each virus CP that may form part of a conserved, structural core is highlighted in yellow. The N-termini of all CP diagrams are left justified, and the helices (H) and strands (S) in ScV-L-A and HvV190S are labeled sequentially. Note that H19 and S31 of ScV-L-A are predicted elements whereas all the others (H1–18 and S1–30) were identified in the crystal structure [30].

Correspondence of these three elements of the unmodified ScV-L-A subunit model with density elements in the HvV190S virion map, though potentially just coincidental, led us to compare the predicted secondary structure of the HvV190S CP with the known secondary structure of ScV-L-A CP (Figure 7), with the goal of deriving a homology model for HvV190S. This procedure seemed warranted given that the ScV-L-A structure contains 24% helix, 21% sheet, and 55% random coil, compared with the prediction of 21% helix, 12% sheet, and 67% random coil for HvV190S. Moreover, the validity of the prediction procedure was at least partially substantiated since the predicted secondary structure for the first 651 aa of ScV-L-A CP (24.4% helix, 20.4% sheet, and 55.2% coil) corresponded almost perfectly with the crystal structure. HvV190S CP has 19 predicted helices (H1–19), which correspond in number but not primary-sequence locations to 18 seen in the ScV-L-A crystal structure, plus one predicted in the missing C-terminal region of ScV-L-A (Figure 7). However, HvV190S CP has only 13 predicted β-strands (S1–13) compared to 30 plus 1 that are found in ScV-L-A (Figure 7).

We next tried to assign predicted secondary structures in HvV190S to the densities that matched between ScV-L-A and HvV190S in our model-fitting analysis described above. Regarding the large, antiparallel β-sheet in ScV-L-A that matches the plate-like density feature in the HvV190S map (Figure 6), two of the main β-strands in this sheet in ScV-L-A (strands 2 and 3; Figure 7) appear to match predicted strands S1 and S2 in HvV190S (aa 30–41 and 50–59); however, the other four β-strands in this sheet in ScV-L-A (strands 10, 18, 25, and 26) have no obvious correspondence in the predictions for HvV190S. Regarding helix H5 in ScV-L-A, which matches tubular density in the HvV190S map, predicted helices in HvV190S that might correspond are H3 (aa 108–122) or possibly H2 (aa 84–98) (Figure 7). Alternatively, the tandem stretch of helices 3, 4, and 5 in ScV-L-A could correspond with the region of HvV190S that contains both predicted helices H2 and H3. Lastly, regarding helix 13, which is the largest helix in ScV-L-A (aa 358–383), it appears to correspond well to two consecutive predicted helices in HvV190S, H11 (aa 369–380) and H12 (aa 385–396). Moreover, helix 13 of ScV-L-A superimposes quite well with a long (∼30 Å) tubular density in the HvV190S map. Given this assignment, helix 12 of ScV-L-A may correspond to predicted helix H10 in HvV190S (aa 350–367), but breaks in density in this and other regions of the HvV190S map made it impossible to determine unambiguously what the CP fold is either locally within the core or elsewhere in the subunit.

The net result of our modeling analysis confirmed a previous suggestion [37] that the CP folds of ScV-L-A and HvV190S differ considerably. Also, we saw no evidence to suggest that flexible fitting of the ScV-L-A model to the HvV190S virion map would improve our interpretations of the data. Regardless, and despite the numerous and obvious differences between the CPs of ScV-L-A and HvV190S, our results show that their capsid structures are related and appear to have retained several elements that have survived their divergent evolutionary paths.

## Discussion

### Other totivirus CPs share a conserved core

As described above, two helix-rich regions in HvV190S CP, encompassing two helices in a 39-aa stretch (H2–3, aa 84–122) and three helices in a 48-aa stretch (H10–12, aa 350–397) appear to correspond, respectively, to the helix 3–5 and helix 12–13 core regions in the crystal structure of each ScV-L-A CP subunit (Figures 6 and 7). This apparent detection of a similar, helical core in these two rather distantly related totiviruses (prototypes of different genera) [17] led us to explore whether other totiviruses may contain this conserved core, even though the overall tertiary structures of the CPs may be quite different. We therefore expanded our analysis of predicted secondary structures to include several other *Totiviridae* members.

Comparisons of the predicted secondary structures of six additional victorivirus CPs revealed dramatic conservation of not just the putative H2–3 and H10–12 core components of HvV190S, but also the primary-sequence distribution of nearly every secondary-structure element in the proteins, including the β-strands (Figure 7). Furthermore, all seven victorivirus CPs that we examined have large C-terminal regions (ranging from 130 to 136 aa long; Figure 7) that are rich in Ala, Gly, and Pro residues and predicted to assume a random-coil structure. This finding is consistent with previous results [17], including that a 130-aa C-terminal region of HvV190S CP is dispensable for VLP assembly [40]. The overall close correspondence among the predicted secondary structures of the seven victorivirus CPs is consistent with the moderate level of sequence identity that each shares with HvV190S, ranging from 62% to 37% (Table S1). Considered together, these findings suggest that all victorivirus CPs are likely to adopt nearly identical tertiary structures and to assemble into very similar, smooth-surfaced, “T = 2” capsids. The T = 2 organization of capsid subunits is a highly conserved feature related to the unique life cycle of dsRNA viruses. These T = 2 capsids organize the replicative complex that is actively involved in genome transcription and replication. Hence, the capsids of all victoriviruses most likely have T = 2 arrangements of its subunits.

Five additional totitviruses, including representatives of three genera whose members infect protozoa (GLV, LRV1, and TVV1) and two unclassified viruses (EbRV1 and IMNV), also appear to include a centrally located stretch of residues with predominantly helical content, which might correspond to the shared core helices of ScV-L-A and HvV190S described above (Figure 7; regions highlighted in yellow). In fact, both LRV1 and EbRV1 CPs contain several regions of predicted secondary-structure elements that lie upstream of the putative H12–13 core helices and form a pattern that closely mimics those observed in victoriviruses. These elements include ones corresponding to the H3–5 and S2–3 elements of ScV-L-A and the H2–3 and S1–2 elements of HvV190S. In addition, the C-termini of LRV1 and EbRV1 CPs are predicted to include a substantial amount of random-coil structure (EbRV-1 more than LRV1). In sum, these findings suggest that the CPs of LRV1 and EbRV1 fold similarly to those of ScV-L-A and HvV190S, and especially to the latter. Further support for the greater similarity to HvV190S includes that the helical-core region of HvV190S shares 25% and 30% sequence identity with the corresponding regions of LRV1 and EbRV1, respectively (Table S2), whereas that region of ScV-L-A shares only 21% and 17% identity with those respective viruses. In addition, a recent phylogenetic analysis of the CP and RdRp sequences of 23 different totiviruses indicated that LRV1 and EbRV1 are more closely related to victoriviruses than they are to any other totiviruses [17]. Interestingly, EbRV1 is also the only other known totivirus outside the genus *Victorivirus* that has an AUGA motif comprising its apparent CP stop and RdRp start codons and therefore might also use a stop–restart strategy to translate its RdRp (GenBank accession number AF356189). Thus, we predict the overall structures of the LRV1 and EbRV1 capsids share significant similarities to the HvV190S capsid reported here.

Our secondary-structure predictions for totiviruses GLV and TVV1, and tentative totivirus IMNV, on the other hand, show fewer specific correspondences with HvV190S or ScV-L-A, or with one another. Although their CPs contain helix-rich regions (highlighted in yellow, Figure 7) that might correspond to the helical-core region represented by H10–12 in HvV190S, this assignment is not as compelling for GLV and TVV1. The presence of fiber complexes at the I5 vertices in IMNV revealed by cryoEM moreover clearly distinguishes IMNV from all other totivirus structures studied to date, though its capsid shell is organized in a manner similar to the HvV190S and ScV-L-A capsids [12]. We thus predict in addition from our analysis here that the capsids of GLV and TVV1 will be found to exhibit some distinctive features, as recently determined cryoEM structures do indeed suggest this to be true (unpublished data).

### Further comparisons of HvV190S virions and VLPs

The capsids of the HvV190S virions and VLPs were shown to be composed of 120 protein subunits arranged in a “T = 2” capsid, with relatively smooth features, consistent with what was observed in previous studies of the empty capsid shell at ∼14-Å resolution [37]. Given that the encapsidated dsRNA mycoviruses never leave the host cell, the primary functions of the capsid as best we know are to protect the genome and replication intermediates and to participate in the replication and transcription of the dsRNA genome [1], [31]. Another important function of the capsid is likely to sequester the dsRNA, which is a potent inducer of host defense mechanisms [41]. Most of these capsids have been shown to contain small pores, through which free nucleotides may enter and newly synthesized plus-strand RNA transcripts may exit during transcription, and through which the dsRNA does not normally exit or cellular proteins such as RNases do not normally enter [39], [42], [43]. Although the possible role of RNA in assembling these capsids remains unknown, it is currently thought that genomic RNA (whether single or double stranded) is not required for particle assembly and packaging of RdRp. Two lines of evidence support this conclusion: First, purified preparations of HvV190S always contain a small fraction of slowly sedimenting, empty capsid component (the 113S component) that packages RdRp but not RNA (Figure S1 B, C; [23], [44]). Second, when virus-free *H. victoriae* protoplasts are transformed with full-length cDNA of HvV190S dsRNA, the integrated cDNA is transcribed and the full-length transcript is translated into CP and RdRp [9], [27]. The CP is assembled into VLPs (VLPc+) that package RdRp but not genomic RNA. VLPc+ sediments at a similar rate to the naturally occurring 113S empty-capsid component when subjected to sucrose density-gradient centrifugation [9], [27].

Close inspections of the equatorial sections of virions and VLPs (Figure 3 C, F) reveal subtle shifts in density positions in certain regions of the capsid, especially near the symmetry axes. To aid in seeing and interpreting these differences, we generated a morph movie in Chimera, highlighting the transition between HvV190S virion and VLP_C+_. The results in particular exhibit expansions of the VLP_C+_ capsid near the I2 and I3 axes concurrent with a contraction at the I5 axes, resembling a “breathing” motion (Movie S1). Since the only known difference in components between the two particle types involves the presence or absence of dsRNA genome, this breathing is likely attributable to contacts between the inner capsid surface and the dsRNA in virions, which are not present in VLP_C+_. Such movements are consistent with points of contact between capsid and genome suggested by densities spanning the sub-capsid space at the I2 axes of virions, which possibly pull the capsid inward at those sites (Figure 3C).

### CP heterogeneity in victorivirus capsids

CP heterogeneity is a property of victoriviruses not shared by other totiviruses [9], [28]. Preparations of the three HvV190S particle types (virion, VLPc+ and VLPc) examined in the current study shared similar amounts of CP forms p88, p83, and p78, with p88 and p78 present in comparable amounts and p83 present in smaller amounts (Figure 1; see also Figure S1B, which demonstrates that the intensity of the Western blot bands correlates well with the intensity of the corresponding Coomassie-stained bands from similar HvV190S and VLPc+ preparations). For simplicity here, we will consider only particles containing p88 and p78 (190S-2; Figure S1A), the predominant type in virion preparations examined (though similar considerations would apply to particles containing p88 and p83). How are the two forms of CP distributed in the capsids? We envision at least four basic, most likely ways in which the uncleaved (p88) and C-terminally cleaved (p78) forms could be arranged. One possible arrangement would have p88 and p78 constituting 60 asymmetric heterodimers, with all p88 subunits in the A position and all p78 subunits in the B position, or vice versa, or with p88 and p78 randomly distributed between A and B positions. A second would have p88 and p78 constituting 30 asymmetric homodimers each, which are randomly distributed in the capsid. A third possible arrangement would have p88 and p78 constituting a random mix of hetero- and homodimers, which are furthermore randomly distributed in the capsid. Lastly, a fourth would have p88 and p78 segregated in different particles. Our current results do not distinguish among these possibilities, although a sorting procedure based on particle mass might have been able to prove the fourth, albeit unlikely possibility. In addition, if the CP C-terminal region (the part missing from p83 and p78) is located in a regular, ordered position within the capsid (see next paragraph for other possibilities), then we might have been able to distinguish subsets of the first possibility in which p88 and p78 are each respectively and consistently located in either the A or the B position. However, although we did not see structural evidence in favor of this consistent segregation of p88 and p78 between A or B positions, we cannot rule out this possibility because the ∼7-Å resolution obtained in this study is likely insufficient to detect these differences.

We can also address where the heterogeneous region of HvV190S, i.e., the C-terminal regions that are present in p88 but absent from p78, are most likely to be located in the particle, in terms of the different capsid surfaces and radii. This region of p88 is predicted to have a random-coil conformation (Figure 7), consistent with its high Ala, Gly, and Pro content, and has also been shown to be dispensable for VLP assembly [40]. Susceptibility to cleavage in this region of p88, to yield p83 and p78, is moreover consistent with its predicted disorder. Whether cleavage occurs pre-or post-assembly is not presently known. To shed some light on this, purified HvV190S virions were treated with chymotrypsin under conditions that result in gradual/partial digestion of bovine serum albumin (Figure S4C). The finding that protease treatment failed to generate additional cleavages in the C-terminal region of CP forms p88 and p83 (Figure S4B) suggests that the C-terminal region is most likely located internally to the CP shell, within the central cavity of the particles in which dsRNA and RdRp molecules are packaged in virions, and thus the cleavages in this region that produced CP forms p83 and p78 that are found in particles most likely occurred before (or during) particle assembly. Furthermore, SDS-PAGE analysis of the same purified HvV190S preparation on a 15% polyacrylamide gel did not reveal the presence of polypeptides of 10 kDa (predicted size of the C-terminal tail; Figure S4A). These results further lend support to the contention that CP proteolytic processing occurs pre-assembly.

### Where is the HvV190S RdRp within particles?

Image analysis of scanned Western blots of purified HvV190S virions have indicated that the CP and RdRp subunits are present at a ratio of ∼65∶1, supporting the notion that each virion contains 120 CP subunits and, on average, two RdRp molecules [4], [24], [39]. An original goal of this study was to locate the HvV190S RdRp within particles. Cryo-reconstruction methods have shown that there are 10–12 RdRp molecules attached to the inner surface of the “T = 2” capsid near the I5 axes in several, large dsRNA viruses, including mammalian orthoreovirus [45]; an aquareovirus [46]; a cypovirus [47], [48]; simian rotavirus [49]; and bacteriophage φ6 [50], [51]. Hence, HvV190S, with just 1–2 copies of RdRp per virion, represents an attractive model system to explore how an RdRp functions in the simplest of the encapsidated dsRNA viruses [9].

By imaging samples of HvV190S virions and two types of VLPs that lack genome, but one of which (VLP_C+_) still contains the RdRp, we hoped to use difference-mapping procedures to locate the RdRp. This effort fell short as described above, however, in that the VLP structures are essentially identical and differ from the virion structure primarily with respect to only small (∼2–3 Å), rigid-body movements of the A and B capsid subunits (Movie S1). No additional density features were observed beyond those already ascribed to the 120 CP subunits or the genome. In the end, this lack of density for the RdRp was not surprising given that all three of the HvV190S cryo-reconstructions were computed with imposed icosahedral symmetry, and hence the signal from a component present only 1–2 copies per particle would be reduced at least 60-fold. Attempts to process the HvV190S image data with lower (fivefold) or no assumed symmetry have yet to yield conclusive clues about where the RdRp is located. This lack of success to date may simply reflect a need for many more particle images (at least 12 times more if only fivefold symmetry averaging is employed or 60 times more if no symmetry averaging is used) to achieve a signal-to-noise ratio close to that obtained in the reconstructions reported here.

Considerations in the preceding two paragraphs include an assumption that the HvV190S RdRp is likely to be anchored to the inner surface of the capsid in a regular, stable manner, but this assumption might not be correct. If the RdRp forms only weak or nonspecific interactions with the capsid, then each RdRp could be oriented differently in each virion or VLP_C+_, and any particle-based averaging procedure would yield a reduced RdRp signal. It is also possible, as suggested previously for HvV190S [37], that because all victorivirus RdRps are expressed as separate, non-CP-fused proteins, they might anchor to the genome but not the capsid. Based on findings with other dsRNA viruses, however, we suspect that the victorivirus RdRp is likely anchored to the capsid close to the I5 axis near the base of the cavity where the end of a newly synthesized RNA transcript might accumulate and then exit if, for example, the proximal tips of the A-subunits rotate away from the axis to create a pore that is large enough to allow the RNA to escape from the particle and enter the host cytoplasm.

## Materials and Methods

### Preparation and purification of HvV190S virions and VLPs

Purified HvV190S virions were isolated from infected *H. victoriae* strain A-9 (ATCC 42018) as previously described [27], [28]. VLPs were isolated from *H. victoriae* strain B-2 (B-2ss, ATCC 42020) that was transformed with plasmid p190S (for VLP_C+_) as described [27], or with a mutant derivative of p190S (p190S/CP) lacking the RdRp ORF (for VLP_C_). Construction of plasmid p190S/CP was based on the previously described transformation/expression vector p190S [27]. A pair of primers, a forward F*se*I-a-F: 5′ GTCTTT*GGCCGGCC* **AGATGTCGGT** 3′ and a reverse primer, CP2608R: 5′ ATATAT*ATCGAT* **TCATTGTCCCTCG** 3′, each containing a restriction site (in italic font) and a stretch of CP sequence (in boldface). The primer pair was used to amplify a small fragment of the CP gene using p190S as a template. Primer F*se*I-a-F contains the restriction site of *Fse*I that is unique and located at nt position 2171 in the CP sequence. A C*la*I restriction site, which is not present in HvV190S, was added to the 3′ end of the CP sequence. The amplified fragment of CP is from nt 2171 to 2608 and was cleaved by *Fse*I and C*la*I and cloned into F*se*I/C*la*I-digested vector p190S, which contains the remaining larger fragment (nt 1–2170) of the CP sequence.

All three particle types were purified from 14-day old stationary cultures grown in potato dextrose broth supplemented with 0.5% (wt/vol) yeast extracts as described [29]. Briefly, clarified fungal extracts were subjected to two cycles of differential centrifugation followed by rate zonal centrifugation in sucrose density gradients (100–400 mg/ml). The major band was then withdrawn with a syringe from the side of the tube and diluted with Buffer A (50 mM Tris-HCl buffer, pH 7.8, containing 5 mM EDTA and 150 mM NaCl). The particles were then concentrated by overnight centrifugation at 40,000 rpm in a Beckman 50Ti rotor and the pellets were resuspended in Buffer A. SDS/PAGE analysis showed that all particle types used in the microscopy studies contained similar amounts of p88, p83, and p78 (Figure 1).

### Electron microscopy of HvV190S

Transmission electron microscopy (TEM) of negatively stained or unstained, vitrified HvV190S samples was performed as described [52]. Negative stain TEM was used to monitor the integrity and homogeneity of all samples. For this, 3.5-µl aliquots of each HvV190S particle type (∼1–10 mg/ml for V and ∼1–5 mg/ml for VLP_C+_ and VLP_C_) were absorbed to continuous carbon grids that had been glow-discharged for ∼25 s in an Emitech K350 evaporation unit and subsequently stained with 1% aqueous uranyl acetate and rinsed with deionized distilled H_2_O. Micrographs were recorded on a 4 K^2^ Gatan CCD camera in an FEI G2 Tecnai (Polara) microscope.

For cryo-EM, a 3.5-µl aliquot of each sample was absorbed to a continuous carbon grid that had been glow-discharged as stated above. An FEI Mark III Vitrobot was used to blot each grid for ∼4 s before plunging it into liquid ethane slush. Frozen grids were then transferred into a pre-cooled, multi-specimen holder, which maintained the specimen at liquid nitrogen temperature. Micrographs were recorded on Kodak SO-163 electron-image film at 200 keV in the Polara microscope under minimal-dose conditions (∼24 e/Å^2^) at a nominal magnification of 59,000. The objective lens defocus settings, and the number of micrographs recorded for each particle type are listed in Table 1.

### Cryo-reconstructions of HvV190S

Micrographs were initially screened by eye to select for ones that exhibited minimal drift and without excessive astigmatism, and where the particle distribution and concentration were adequate. Acceptable micrographs were digitized at 6.35-µm intervals on a Nikon Supercoolscan 8000 microdensitometer. Programs AUTO3DEM [53] and RobEM (http://cryoEM.ucsd.edu/programs.shtm) were used to process the micrographs. This included boxing out individual particles (501^2^ pixels), followed by apodization, normalization, and linear gradient correction of these data. For the virion data set, only particles that appeared to be full (*i.e.* containing the entire genome) were boxed and analyzed further. Program ctffind3 was used to estimate the defocus value of each micrograph [54]. Next, the random-model computation procedure [55] was used to generate an initial 3D reconstruction at ∼30-Å resolution from 150 particle images for each specimen. Initial reconstructions obtained in this way served as starting models for the AUTO3DEM program (version 4.01.07, http://cryoem.ucsd.edu/programs.shtm), which were used to determine and refine the origin and orientation parameters for all particles in each data set. This process typically required 20 iterations until no further improvement in the resolution of the current reconstruction was achieved. The resolution limit attained for each cryo-reconstruction was estimated by the Fourier Shell Correlation method (Figure S2) and applying a conservative 0.5 threshold criterion (FSC_0.5_) [56].

During the course of this study, new functionality was added to AUTO3DEM that helped improve the quality of the final HvV190S reconstructions. First, a new option for re-centering particles was added to the *autopp* program used for batch processing. Second, two new search modes were added to the *PO^2^R* refinement program.

Accurate estimates of particle orientation parameters rely on particles being properly centered in the box window, which is rarely if ever perfect for any particle picking method (either manual or automatic). Hence, after the first round of 20 iterations of origin and orientation refinement finished and the estimate for the origin of each particle did not change appreciably, all particles were re-extracted from the original set of digitized micrographs to assure that the center of the particle lies within one half pixel of the center of the new box window. This reboxing procedure was automated by implementing an *ad hoc* option in *autopp*.

On occasion, for some particles the template-based orientation refinement procedure can get trapped in a local minimum that does not correspond to the optimal solution. Hence, to help assure that the correct orientation is assigned to each particle, a global orientation search is recomputed for all particle images after repeated cycles of PO^2^R refinement fail to further improve the reconstruction. This global search, which is handled by the *porg* option in AUTO3DEM, covers the entire icosahedral asymmetric unit at an angular step interval defined by the user (two degrees in this study), in each of the three Euler angles. It differs from the initial global search procedure normally performed with PPFT, primarily because it makes use of the most recent and presumably higher resolution reconstruction as a template, and PO^2^R is used for the search. The advantage is that the algorithm implemented in PO^2^R is more accurate than that in PPFT, though PO^2^R involves a much higher computational load.

Given that the HvV190S image data were acquired over many microscopy sessions, the magnifications of the different micrographs varied enough to warrant an additional image processing step, implemented as the *porm* option in AUTO3DEM. Here, the relative magnification of each micrograph was estimated (±2% range scanned at 0.1% intervals) by determining the average for all particles in each micrograph of the radial scale factor that maximized the correlation between each particle image with its corresponding projection of the reconstruction. Based on this analysis, the pixel size assigned to each micrograph was adjusted to assure that the 2D particle transforms were properly scaled and merged in program P3DR [57] to form the 3D Fourier transform and subsequent 3D density map.

Each time any of the above procedures was performed (reboxing, *porg*, or *porm*), an additional 20 iterations of local refinement were carried out with PO^2^R. This process led to an overall gain in resolution for the HvV190S virion reconstruction from 7.8 to 7.1 Å. To this point, all procedures were carried out with reconstructed density maps computed with image data that had been corrected to compensate for both amplitude and phase effects of the microscope contrast transfer function as described [58]. To aid in our analysis and interpretation of the HvV190S capsid structure, the final reconstructed density maps were only computed with phase flipping [59]. The pixel size for the virion map was calibrated as described below, but not for either of the VLP density maps. These were radially scaled against the virion map in RobEM as described [52], [60]. Features at high spatial frequencies in the virion map were enhanced by application of an inverse temperature factor of 200 Å^2^, which was chosen heuristically [61]. We used the program *bampweigh* in Bsoft to scale the radially-averaged Fourier amplitude spectra for the two VLP density maps to the spectrum computed from the sharpened virion map [62]. RobEM and Chimera [63] were used to visualize and analyze all three density maps. The thresholds used to render the density maps were set to give a capsid volume consistent with 120 copies of an 81-kDa protein.

### Calibration of the pixel size of the HvV190S virion 3D reconstruction

An HvV190S virion sample was mixed with a solution at similar concentration of a known standard, HK97 prohead II [38] to determine the absolute size of HvV190S. A 3.5-µL aliquot of this mixture was vitrified and cryo-EM was performed as described above for all other HvV190S samples. Six of the 54 micrographs that were recorded contained enough HvV190S and HK97 prohead II particles to enable us to compute a separate 3D reconstruction of each particle type in each micrograph. Radial density plots were computed for all twelve reconstructions [64] as well as from a density map generated from the crystal structure of the HK97 prohead II (PDB ID 3E8K [38]). The known scale of the HK97 density plot derived from the crystal structure provided an absolute measure of the pixel size for each HK97 reconstruction and hence the magnification of each of the six micrographs (Table S3). These tests showed that the average magnification of the six micrographs was 59,318, which is within 0.5% of the nominal magnification (59,000) of the Polara microscope. Knowledge of the calibrated magnification of each micrograph allowed us to define the absolute scale of the HvV190S reconstruction. This, for example, showed that the highest average radial density in the HvV190S capsid occurred at a radius of 183.5 Å (Table S3).

### Segmentation of the HvV190S virion electron density map

The molecular boundaries of the A and B subunits that comprise each asymmetric unit of the HvV190S capsid were delineated by means of an iterative segmentation protocol specifically developed for this task. All the steps during this process, beginning with the final reconstructed map represented as a surface rendering, were performed using tools available in Chimera. The threshold used to render the HvV190S density map was chosen to account for the total expected molecular mass of the 120-subunit capsid (9,720 kDa). The *Segger* tool in Chimera [65] was used with its default values to initially segment the map into small “regions”, and only those regions identified as belonging to a single asymmetric unit (e.g. as defined by the symmetry axes) were considered for further analysis. Visual inspection of the density map clearly showed that there was a set of similar features on the outer surface of each subunit and this enabled us to manually group the *Segger* defined regions near these features into two separate subvolumes. Based on this initial estimate, these subvolmes were extended and refined in an iterative manner. At each iteration, this involved masking out densities in the reconstruction (*Segger*) according to the current definition of the subvolumes and then modeling these densities as a grid of markers (*meshmol*), initially at a coarse (two voxel) spacing and later at a finer (one voxel) spacing. Next the closest, symmetry-related copies of each grid model (3 for A and 4 for B) were generated (*sym*) and converted into separate density models (*molmap*). Finally, these density models were visually analyzed, and newly segmented subvolumes were obtained by refining their boundaries. Refinement involved the imposition of two constraints on the models. First, the A and B subunits were assumed to adopt similar tertiary structures. Hence, at each iteration, we superimposed and compared the current solution for each subunit, to verify their agreement. Density features that were present in only one subunit were removed and temporarily left unassigned. As a second constraint, we required that a model of the capsid comprised of both subunits would account for all the capsid density in the reconstructed map. We verified this constraint by imposing icosahedral symmetry on the two subvolumes, and manually inspected all inter-subunit interfaces to identify inconsistencies in our assignments. For example, regions of overlap required re-segmentation, and unassigned density in the original map, which apparently belonged to the A or B subunit, was included in the appropriate subvolume. Following this approach, the boundaries of the A- and B-subunits were iteratively refined, until these constraints were fully satisfied.

### Fit of ScV-L-A CP X-ray structure into the HvV190S virion density map

The crystal structure of the ScV-L-A virus CP (PDB 1M1C) [30] was manually docked as a rigid body into each of the A and B subunit maps that were segmented as described above. The Chimera *fit_in_map* tool was then used to refine independently the rigid body fit of the ScV-L-A model into each subunit. The normalized correlation value of the final fitting was 0.37 for both subunits, as provided by Chimera.

### Locating putative α-helices in the HvV190S A and B subunit density maps

Density features that could be assigned to α-helical segments were annotated in the A- and B- subunits of the virion as obtained by the segmentation procedure. The locations and lengths of the helices were predicted automatically using the VolTrac algorithm [66] implemented in the Sculptor graphics software [67]. Following the procedure outlined in [66], a Gaussian-weighted local normalization was applied to the densities, using a standard deviation equal to half the nominal resolution. Then the prediction algorithm was executed with the target number of helices set equal to 20. All program parameters were set to the default values, except the expansion threshold was set to 75%. Since the first 20 helices ranked by the prediction algorithm differed for the two subunits, we examined visually all the solutions found by the program (62 and 59 for A and B, respectively), and we retained only those that were common to both (33 helices).

### Secondary structure prediction of the coat proteins of HvV190S and other totiviruses

We used PSIPRED and default parameter values (http://bioinf.cs.ucl.ac.uk/psipred/) to predict the secondary structures of the CPs of HvV190S (p88, GenBank accession number U41345.2) and eleven other viruses in the family *Totiviridae*. These include (along with the genera to which they are assigned and their GenBank accession numbers) *Victorivirus* BfTV1 (Botryotinia fuckeliana totivirus 1, AM491608), CeRV-1 (Chalara elegans RNA virus 1, AY561500), CmRV (Coniothyrium minitans RNA virus, AF527633), GaRV-L1 (Gremmeniella abietina RNA virus L1, AF337175), SsRV1 (Sphaeropsis sapinea RNA virus 1, AF038665), and SsRV2 (Sphaeropsis sapinea RNA virus 2, AF039080); unassigned EbRV1 (Eimeria brunetti RNA virus 1, AF356189) and IMNV (penaeid shrimp infectious myonecrosis virus, AY570982); *Giardiavirus* GLV (Giardia lamblia virus, L13218); *Leishmaniavirus* LRV1-4 (Leishmania RNA virus 1–4, U01899); and *Trichomonasvirus* TVV1 (Trichomonas vaginalis virus 1, GU08999).

### Accession numbers

The density maps of the cryo-reconstructions of the HvV190S virion, VLP_C+_, and VLP_C_ have been deposited in the Electron Microscopy Data Bank at the European Bioinformatics Institute with accession codes EMD-26556, EMD-26557, and EMD-26558, respectively. GenBank accession numbers for the capsid proteins of thirteen related viruses in the family *Totiviridae* are provided in Tables S1 and S2.

## Supporting Information

Figure S1 **SDS-polyacrylamide gel electrophoresis of HvV190S virions and Western blot analysis.** (**A**) SDS-polyacrylamide analysis of the 190S-1 and 190S-2 forms following two cycles of sucrose density gradient centrifugation; capsids of 190S-1 contain similar amounts of p83 and p88, whereas those of 190S-2 contain comparable amounts of p78 and p88. (**B**) Capsid composition of HvV190S virions, associated empty capsid (HvV113S component), and VLPc+ as determined by Coomassie Blue staining (left) and Western blot analysis using an HvV190S CP-specific antiserum (right). (**C**) The naturally occurring empty capsid fraction (113S component) packages RdRp. Samples of the same preparations shown in panel (B) were analyzed by Western blotting using an HvV190S RdRp-specific antiserum. HvV113S represents a small fraction of slower-sedimenting empty capsid component (designated as the 113S component based on its sedimentation value of 113S), and is always detected in association with purified HvV190S virions.
(TIF)

Figure S2 **Fourier Shell Correlation curves.** The correlation curves calculated for the HvV190S virion, VLP_C+_, and VLP_C_ intersect the threshold 0.5 (0.143) at 7.1 (6.8), 7.5 (7.1) and 7.6 (7.2) Å, respectively. See Table 1 for relevant reference citations for these calculations.
(TIF)

Figure S3 **Radial density plots.** Radial density plots [64] calculated from the HvV190S virion, VLP_C+_, and VLP_C_ cryo-reconstructions for radii between 10 and 255 Å. The dsRNA genome in virions is organized into five, roughly spherical shells that appear as distinct peaks in the radial density plot, centered at radii from a high of ∼143 Å (first shell) to a low of ∼23 Å (fifth shell).
(TIF)

Figure S4 **Effect of in vitro protease treatment on purified HvV190 virions.** (**A**) Search for polypeptides 10 kDa or smaller generated by proteolytic processing of p88 of HvV190S, VLPc+, and VLPc after particle assembly. Purified preparation of HvV190S virions, VLPc+, and VLPc as well as total proteins were subjected to SDS-PAGE on a 15% polyacrylamide gel and Western blot analysis using a CP-specific antiserum. A total of 5 µg of HvV190S virions, VLPc+, and VLPc were analyzed as well as 30 µg of total proteins extracted from HvV190S-infected fungal host or from host transformed with plasmids p190S (for VLPc+) or p190S/CP (for VLPc). For extracting total proteins, *H. victoriae* cultures containing HvV190S or transformed with p190S and p190S/CP were grown on cellophane-covered potato dextrose agar medium (PDA) supplemented with 0.5% (wt/vol) yeast extracts. Mycelium was collected from 10-day old cultures and ground in phosphate buffered saline (PBS) buffer, pH 7.4, supplemented with 1 mM protease inhibitor phenylmethylsulfonyl fluoride (PMSF). The homogenate was centrifuged for 15 min at 10,000 rpm at room temperature. Protein concentration in the supernatant was determined with Bio-Rad protein assay reagent. A polypeptide of 10 kDa was not detected, which suggests that processing did not occur post-assembly. (**B**) In vitro protease treatment of purified virions and VLPs. α-chymotrypsin from bovine pancreas (C-7762 from Sigma). (**C**) Bovine serum albumin (BSA) was used as positive control substrate for assessing the proteolytic activity of α-chymotrypsin. All the test substrates including BSA were used at a concentration of 5.2 µM and the final enzyme concentration was 5 µM. For each reaction, 34 µl BSA (1 µg/µl), or 20 µl virions (2.3 µg/µl) was mixed with 0.5 µl chymotrypsin (25 µg/µl), 2 µl Tris-HCl (500 mM, pH 7.8) and the volume was adjusted to 100 µl with sterile water. A blank control consisting of only the substrates, Tris-HCl buffer and sterile water (buffer only) was also included. Reactions were incubated at 25°C for 5 min, 10 min, 15 min, and 20 min, or 30 min separately except that the blank was incubated for 20 or 30 min. Following incubation, PMSF was added to each reaction at a final concentration of 1 mM. After incubation, samples were analyzed on 7.5% SDS-PAGE gel, and a non-treated substrate was included for each analysis. Following electrophoresis, the gels were Commassie-stained for 30 min and destained. In vitro protease treatment failed generate additional cleavages in the C-terminal region suggesting that cleavages in this region that produced CP forms p83 and p78 most likely occurred before (or during) particle assembly.
(TIF)

Movie S1 **Conformational differences between the HvV190S virion and VLP_C+_ capsid structures.** Differences are highlighted by means of an animation produced with the Morph Map tool and default values in Chimera [63]. 3D density maps of the virion and VLP_C+_ cryo-reconstructions, each band-limited to 9.0-Å resolution and with the front-half removed (also genome density in the virion) to reveal external as well as internal features in the capsid shell, served as the initial and final data sets. The capsid undergoes subtle, coordinated conformational changes akin to “breathing” in response to loss of genome, with regions near the two- and threefold axes expanding and near the fivefold axes contracting during the transition from virion to VLP_C+_. These conformational changes suggest that attractive and repulsive electrostatic forces govern interactions between the genome and the inner surface of the capsid. Close inspection of the animation shows that some regions of the capsid undergo little if any conformational change between the two structures.
(MOV)

Movie S2 **Segmented A-subunit of HvV190S virion and secondary-structure analysis.** This animation, produced using Chimera [63] shows the result of a rigid-body fit of the ScV-L-A A-subunit atomic model, and the resulting locations of its α-helices are compared with the positions of the α-helices predicted from the HvV190S segmented density (see Materials and Methods and the Figure 6 legend for additional details).
(MP4)

Table S1 **Sequence comparisons between the capsid protein of HvV190S and that of six other victoriviruses and six related viruses in the family** ***Totiviridae*** **.** The predicted secondary structures of the capsid proteins of thirteen totiviruses suggest that they have a moderate level of sequence identity. BfTV1 is most closely related to HvV190S as their capsid proteins share 62% sequence identity.
(DOCX)

Table S2 **Percent sequence identity between HvV190S CP conserved helical region (CHR) and the corresponding region in selected victoriviruses and related viruses in the family** ***Totiviridae*** **.** The percent sequence identity was calculated for twelve totiviruses for the CHR and compared to that of HvV190S. Similar to what was found for the complete percent sequence identity over the entire capsid protein, BfTV1 had the highest value (56%), compared to the average sequence identity (31%) when compared to HvV190S.
(DOCX)

Table S3 **Pixel size calibration statistics for HvV190S virion 3D reconstruction.** The pixel size and magnification for each micrograph recorded from the vitrified, mixed sample (HvV190S virions and HK97 prohead II particles) were determined by measuring the peak position of the radial density plot of each HK97 3D reconstruction and calibrating this against the corresponding peak position in the radial density plot computed from the HK97 crystal structure after low-pass filtering to 30-Å resolution was applied. This calibration showed that the radial density plots of the six separate HvV190S virion cryo-reconstructions peaked at an average radius of 183.5±0.7 Å. This value, in turn, allowed us to accurately define the pixel size of the final 7.1-Å resolution reconstruction of the HvV190S virion that was computed from 20,904 virion images (Table 1).
(DOCX)
