# Supplementary figures and images for: Three-dimensional Structure of Victorivirus HvV190S Suggests Coat Proteins in Most Totiviruses Share a Conserved Core
Source: PLoS Pathog. 2013 Mar 14;9(3):e1003225. doi: 10.1371/journal.ppat.1003225 (PMC3597494; doi:10.1371/journal.ppat.1003225)

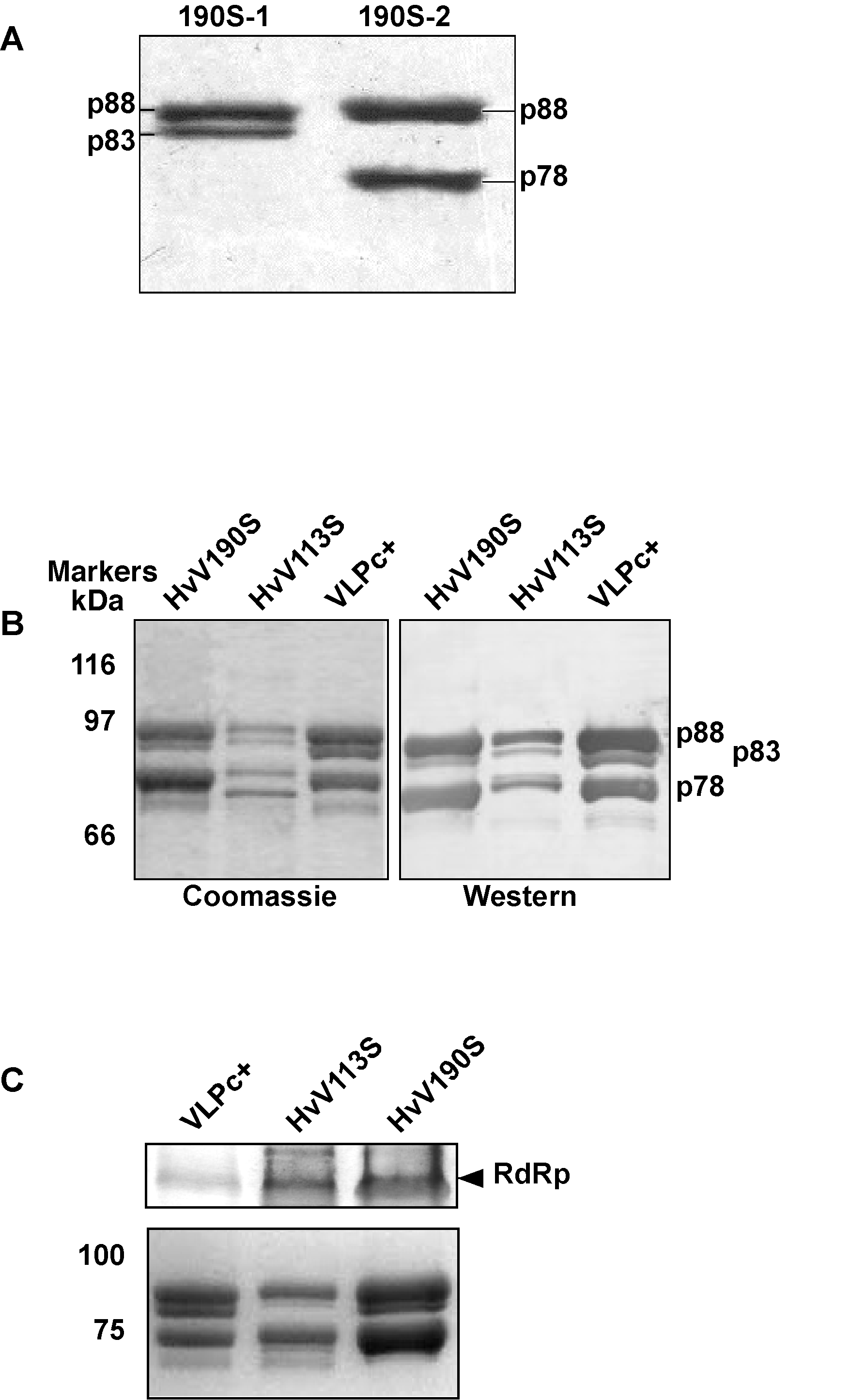

Supplement: Figure S1 — SDS-polyacrylamide gel electrophoresis of HvV190S virions and Western blot analysis. (A) SDS-polyacrylamide analysis of the 190S-1 and 190S-2 forms following two cycles of sucrose density gradient centrifugation; capsids of 190S-1 contain similar amounts of p83 and p88, whereas those of 190S-2 contain comparable amounts of p78 and p88. (B) Capsid composition of HvV190S virions, associated empty capsid (HvV113S component), and VLPc+ as determined by Coomassie Blue staining (left) and Western blot analysis using an HvV190S CP-specific antiserum (right). (C) The naturally occurring empty capsid fraction (113S component) packages RdRp. Samples of the same preparations shown in panel (B) were analyzed by Western blotting using an HvV190S RdRp-specific antiserum. HvV113S represents a small fraction of slower-sedimenting empty capsid component (designated as the 113S component based on its sedimentation value of 113S), and is always detected in association with purified HvV190S virions. (TIF) [file ppat.1003225.s001.tif]

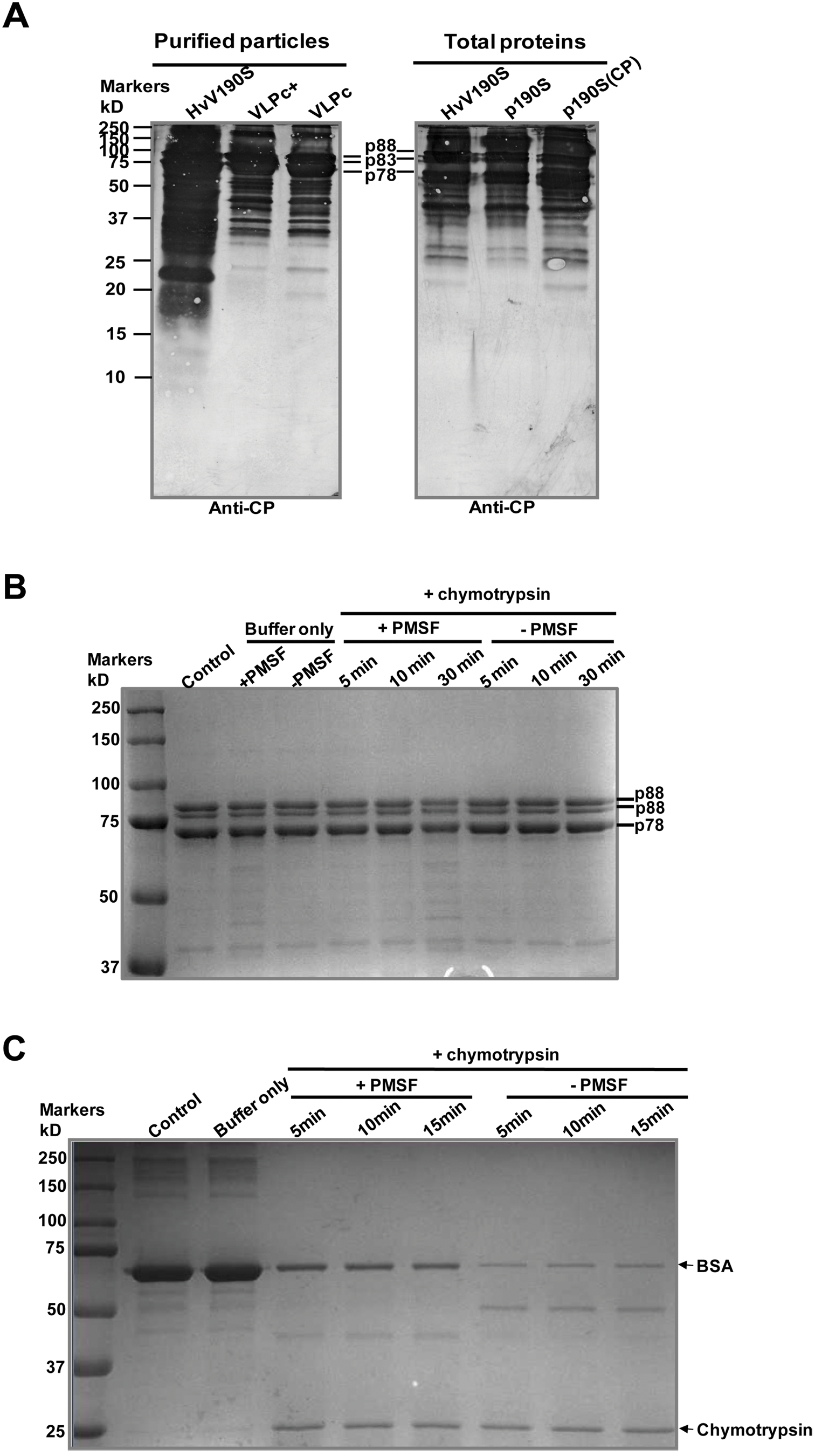

Supplement: Figure S4 — Effect of in vitro protease treatment on purified HvV190 virions. (A) Search for polypeptides 10 kDa or smaller generated by proteolytic processing of p88 of HvV190S, VLPc+, and VLPc after particle assembly. Purified preparation of HvV190S virions, VLPc+, and VLPc as well as total proteins were subjected to SDS-PAGE on a 15% polyacrylamide gel and Western blot analysis using a CP-specific antiserum. A total of 5 µg of HvV190S virions, VLPc+, and VLPc were analyzed as well as 30 µg of total proteins extracted from HvV190S-infected fungal host or from host transformed with plasmids p190S (for VLPc+) or p190S/CP (for VLPc). For extracting total proteins, H. victoriae cultures containing HvV190S or transformed with p190S and p190S/CP were grown on cellophane-covered potato dextrose agar medium (PDA) supplemented with 0.5% (wt/vol) yeast extracts. Mycelium was collected from 10-day old cultures and ground in phosphate buffered saline (PBS) buffer, pH 7.4, supplemented with 1 mM protease inhibitor phenylmethylsulfonyl fluoride (PMSF). The homogenate was centrifuged for 15 min at 10,000 rpm at room temperature. Protein concentration in the supernatant was determined with Bio-Rad protein assay reagent. A polypeptide of 10 kDa was not detected, which suggests that processing did not occur post-assembly. (B) In vitro protease treatment of purified virions and VLPs. α-chymotrypsin from bovine pancreas (C-7762 from Sigma). (C) Bovine serum albumin (BSA) was used as positive control substrate for assessing the proteolytic activity of α-chymotrypsin. All the test substrates including BSA were used at a concentration of 5.2 µM and the final enzyme concentration was 5 µM. For each reaction, 34 µl BSA (1 µg/µl), or 20 µl virions (2.3 µg/µl) was mixed with 0.5 µl chymotrypsin (25 µg/µl), 2 µl Tris-HCl (500 mM, pH 7.8) and the volume was adjusted to 100 µl with sterile water. A blank control consisting of only the substrates, Tris-HCl buffer and sterile water (buffer only) was also in [file ppat.1003225.s004.tif]
