# Supplementary material for: Three-dimensional Structure of Victorivirus HvV190S Suggests Coat Proteins in Most Totiviruses Share a Conserved Core
Source: PLoS Pathog. 2013 Mar 14;9(3):e1003225. doi: 10.1371/journal.ppat.1003225 (PMC3597494; doi:10.1371/journal.ppat.1003225)
Supplement: Table S1 — Sequence comparisons between the capsid protein of HvV190S and that of six other victoriviruses and six related viruses in the family Totiviridae . The predicted secondary structures of the capsid proteins of thirteen totiviruses suggest that they have a moderate level of sequence identity. BfTV1 is most closely related to HvV190S as their capsid proteins share 62% sequence identity. (DOCX) [file ppat.1003225.s007.docx]

**Table S1. Sequence comparisons between the capsid protein of HvV190S and that of six other victoriviruses and six related viruses in the family *Totiviridae***

| Virus Species | *Virus Genus* | Genbank  accession # | CP length (aa) | Sequence identity^b^ | E value^b^ |
| --- | --- | --- | --- | --- | --- |
| HvV190S | *Victorivirus* | U41345 | 772 | 100.0 | 0.0 |
| BfTV1 | *Victorivirus* | AM491608 | 765 | 62.0 | 0.0 |
| SSRV1 | *Victorivirus* | AF038665 | 776 | 49.9 | 1.6E-159 |
| CeRV1 | *Victorivirus* | AY561500 | 770 | 49.1 | 2.8E-146 |
| SsRV2 | *Victorivirus* | AF039080 | 789 | 38.9 | 1.4E-91 |
| GaRV-L1 | *Victorivirus* | AF337175 | 776 | 38.3 | 3.7E-89 |
| CmRV | *Victorivirus* | AF527633 | 775 | 37.0 | 5.6E-89 |
| LRV1-1 | *Leishmaniavirus* | M92355 | 741 | 27.9 | 9.2 E-7 |
| EbRV1 | Unclassified | AF356189 | 770 | 26.9 | 3.0E-36 |
| ScV-L-A* | *Totivirus* | J04692 | 680 | NSH | NSH |
| TVV-1* | *Trichomonasvirus* | U08999 | 673 | NSH | NSH |
| IMNV* | Unclassified | AY570982 | 908 | NSH | NSH |
| GLV* | *Giardiavirus* | L13218 | 886 | NSH | NSH |

^a^ Abbreviations: HvV190S, Helminthosporium victoriae virus 190S; SsRV1, Sphaeropsis sapinea RNA virus 1; CeRV1, Chalara elegans RNA virus 1; SsRV2, Sphaeropsis sapinea RNA virus 2; GaRV-L1, Gremmeniella abietina RNA virus L1; BfTV1, Botryotinia fuckeliana totivirus 1; LRV1-1, Leishmania RNA virus 1-1; EbRV1, Eimeria brunetti RNA virus 1; ScV-L-A, Saccharomyces cerevisiae virus L-A ; TVV1, Trichomonas vaginalis virus 1; IMNV, penaeid shrimp infectious myonecrosis virus; GLV, Giardia lamblia virus.

^b^ Identities and E values comparing the entire capsid proteins were obtained from PSI-Search results (EMBL-EBI; <http://www.ebi.ac.uk>). Similar values were obtained using the [NCBI](http://www.ncbi.nlm.nih.gov/)/ [BLAST](http://blast.ncbi.nlm.nih.gov/Blast.cgi?CMD=Web&PAGE_TYPE=BlastHome)/ blastp suite.

*The CPs of these four totiviruses lack significant sequence identities with HvV190S CP, as determined by [NCBI](http://www.ncbi.nlm.nih.gov/)/ [BLAST](http://blast.ncbi.nlm.nih.gov/Blast.cgi?CMD=Web&PAGE_TYPE=BlastHome)/ blastp suite. NSH, no significant hits.
