# Supplementary material for: Three-dimensional Structure of Victorivirus HvV190S Suggests Coat Proteins in Most Totiviruses Share a Conserved Core
Source: PLoS Pathog. 2013 Mar 14;9(3):e1003225. doi: 10.1371/journal.ppat.1003225 (PMC3597494; doi:10.1371/journal.ppat.1003225)
Supplement: Table S2 — Percent sequence identity between HvV190S CP conserved helical region (CHR) and the corresponding region in selected victoriviruses and related viruses in the family Totiviridae . The percent sequence identity was calculated for twelve totiviruses for the CHR and compared to that of HvV190S. Similar to what was found for the complete percent sequence identity over the entire capsid protein, BfTV1 had the highest value (56%), compared to the average sequence identity (31%) when compared to HvV190S. (DOCX) [file ppat.1003225.s008.docx]

**Table S2. Percent sequence identity between HvV190S CP conserved helical region (CHR) and the corresponding region in selected victoriviruses and related viruses in the family *Totiviridae***

| Virus ^a^ | Genebank accession # | CHR^b^ (aa) | Overlap^c^ (aa) | % identity^c^ |
| --- | --- | --- | --- | --- |
| HvV190S | U41345 | 48 | 48 | 100 |
| BfTV1 | AM491608 | 49 | 48 | 56 |
| CeRV1 | AY561500 | 43 | 34 | 47 |
| SsRV1 | AF038665 | 46 | 39 | 36 |
| GaRV-L1 | AF337175 | 52 | 31 | 32 |
| SsRV2 | AF039080 | 51 | 45 | 29 |
| CmRV | AF527633 | 46 | 37 | 22 |
| EbRV1 | AF356189 | 52 | 20 | 30 |
| LRV1-1 | M92355 | 47 | 24 | 25 |
| ScV-L-A | J04692 | 38 | 21 | 29 |
| IMNV | AY570982 | 46 | 27 | 26 |
| TVV1 | U08999 | 65 | 28 | 21 |
| GLV | L13218 | 36 | 28 | 18 |

^a^ Abbreviations: see Table S1

^b^ Length of conserved helical region (shown as yellow highlighted regions in Figure 7)

^c^ Length of overlap as determined by SIM (<http://web.expasy.org/sim/>).
