# Supplementary material for: Three-dimensional Structure of Victorivirus HvV190S Suggests Coat Proteins in Most Totiviruses Share a Conserved Core
Source: PLoS Pathog. 2013 Mar 14;9(3):e1003225. doi: 10.1371/journal.ppat.1003225 (PMC3597494; doi:10.1371/journal.ppat.1003225)
Supplement: Table S3 — Pixel size calibration statistics for HvV190S virion 3D reconstruction. The pixel size and magnification for each micrograph recorded from the vitrified, mixed sample (HvV190S virions and HK97 prohead II particles) were determined by measuring the peak position of the radial density plot of each HK97 3D reconstruction and calibrating this against the corresponding peak position in the radial density plot computed from the HK97 crystal structure after low-pass filtering to 30-Å resolution was applied. This calibration showed that the radial density plots of the six separate HvV190S virion cryo-reconstructions peaked at an average radius of 183.5±0.7 Å. This value, in turn, allowed us to accurately define the pixel size of the final 7.1-Å resolution reconstruction of the HvV190S virion that was computed from 20,904 virion images (Table 1). (DOCX) [file ppat.1003225.s009.docx]

# Table S3. Pixel size calibration statistics for HvV190S virion 3D reconstruction

| **M^1^** | **Defocus^2^** | **HK97 Particles^3^** | **HK97**  **Resolution^4^** | **Radius^5^** | **Pixel Size^6^** | **Calibrated Mag.** | **HvV190S Particles^7^** | **HvV190S Resolution^4^** | **Radius^8^** |
| --- | --- | --- | --- | --- | --- | --- | --- | --- | --- |
| 1 | 2.36 μm | 99 | 29 Å | 211.50 | 1.073 | 59,180 | 51 | 38 Å | 183.26 |
| 2 | 2.47 μm | 97 | 28 Å | 211.50 | 1.073 | 59,180 | 43 | 42 Å | 183.00 |
| 3 | 3.15 μm | 67 | 33 Å | 211.25 | 1.075 | 59,070 | 31 | 54 Å | 183.21 |
| 4 | 2.32 μm | 142 | 26 Å | 210.00 | 1.081 | 58.742 | 36 | 42 Å | 184.30 |
| 5 | 3.19 μm | 53 | 32 Å | 210.50 | 1.078 | 58,905 | 45 | 37 Å | 184.40 |
| 6 | 3.20 μm | 93 | 31 Å | 211.25 | 1.075 | 59,070 | 27 | 43 Å | 182.67 |

**^1^** Micrograph number

**^2^** Objective lens underfocus value

**^3^** Number of particle images included in each HK97 Prohead II 3D reconstruction

**^4^** Estimate of resolution achieved in 3D reconstruction based on FSC_0.5_ criterion [[56](#_ENREF_56)]

**^5^** Radius in pixels at which the average density is highest for the HK97 Prohead II reconstruction

**^6^** Pixel size based on knowledge that the peak of the radial density plot computed from the HK97 Prohead II crystal structure (PDB ID 3E8K) [38] occurs at a radius of 227 Å.

**^7^** Number of boxed particle images included in each HvV190S 3D reconstruction

**^8^** Radius (in Å) at which the average density is highest for the HvV190S virion reconstruction
